# Emotional Well-Being and Traditional Cypriot Easter Games: A Qualitative Analysis

**DOI:** 10.3389/fpsyg.2021.613173

**Published:** 2021-09-24

**Authors:** Christiana Koundourou, Markella Ioannou, Chara Stephanou, Maria Paparistodemou, Theodora Katsigari, Georgios Tsitsas, Kyriaki Sotiropoulou

**Affiliations:** Department of Psychology, School of Health Sciences, Neapolis University Pafos, Paphos, Cyprus

**Keywords:** emotional well-being, tradition, Cyprus, Easter Games, qualitative analysis

## Abstract

The aim of the current study is to examine the effect of the Traditional Easter Games of Cyprus on the emotional well-being of the participants. Data were collected using a qualitative analysis. It consisted of interviews from 51 participants aged 32–93 years old, and observations were made from audiovisual material of the Traditional Cypriot Easter Games being played by a sample of 20 children aged 6–14 years old and 43 adults aged 18–65 years old. Demographic data were collected by using interviews and analyzed using IBM SPSS program. The observations of the audiovisual material focused on the emotions of the participants and were grouped into prevailing and secondary emotions according to frequency and duration. The results indicate that games produce emotions such as joy, excitement, and euphoria. Emotions such as embarrassment, frustration, and anger were also observed occasionally, specifically in situations of competitiveness and defeat. In addition, the differences and similarities between adults and children were recorded. The findings of the present study extend previous work by demonstrating the positive impact of the traditional games on children’s and adult’s emotional well-being.

## Introduction

### Emotional Well-Being

Feeling good about one’s life may be a core asset in one’s mental and physical health, which, according to the new trend of Positive Psychology, is better described by the term of *emotional* or *subjective well-being.* Emotional well-being is a theoretical multidimensional construct, which among others includes experiencing positive emotions and a sense of fulfilment, satisfaction, and meaning in life (Houben et al., 2015; Pezirkianidis and Stalikas, 2020).

Following Seligman’s innovating model of well-being, which is being recognized by the “PERMA” acronym, well-being is more than just a philosophical idea and it is better explained, and even measured, under the form of five elements (Seligman, 2011): *Positive emotions (P), Engagement (E), positive Relationships (R), Meaning (M), and Achievement (A). Positive emotions* are to describe one’s happiness and what boosts optimism and hope in life, such as pleasure, joy, ecstasy, comfort, warmth, fun, and so on (Kun et al., 2017). *Engagement (E)* is a sense of thrill in being occupied with a pleasant task, or else described as a new state of mind, a sense of “flow,” being carried away and being absorbed by something, or a sense of almost losing consciousness in it. *Positive Relationships (R)* are about supportive, loving, and secure connections with family, peers, friends, and all kinds of social cycles, while *Meaning (M)* and *Achievement (A)* is a state of being productive and insisting in fulfilling one’s personal dreams, meeting challenges, and accomplishing goals (Shoshani and Slone, 2017).

Emotions also resemble moods. Yet emotions differ from moods, in that emotions refer to personally meaningful circumstances (i.e., they have an object), are typically short-lived, and occupy the foreground of consciousness. In contrast, moods are typically free-floating or objectless and more long-lasting occupy the background of consciousness (Lewis, 2008; Tugade et al., 2014).

### Traditional Games

Studies on traditional games show that games and sports were an integral part of humanity (Gelisli and Yazici, 2015; Harteveld and Suarez, 2015; Asmara and Syobar, 2018; Türkmen and Useev, 2019; Fitri et al., 2020). Through the years, different games appeared depending on the climate and geography of each region (Dehkordi, 2017; Fitri et al., 2020). Games related with religion and cultural celebrations contribute to the acquisition of intimacy of individuals with their environment (Groll et al., 2015; Türkmen and Useev, 2019; Fitri et al., 2020) and appear to be a reflection of the society that created them, a “mirror of society” as it is characterized by a study of Nefil et al. (2021). Traditional games are part of cultural heritage (Türkmen and Useev, 2019; Fitri et al., 2020; Luchoro-Parrilla et al., 2021) and they enable individuals to understand and incorporate the moral values passed on from previous generations (Aisyah, 2017; Popeska and Jovanova-Mitkovska, 2017). In addition to this, play can teach children to reduce their egocentrism, appreciate individual differences, and respect the rights of others (Fitri et al., 2020). MacLean et al. (2017) also argue that higher levels of well-being are measured in cultures where cultural values are still preserved, which is an important argument for the perpetuation of Traditional Games.

### Traditional Games and Emotional Well-Being

Additional studies support that traditional games promote physical and emotional well-being and individuals are overwhelmed by satisfaction, energy, euphoria, and joy, enabling the player to be more creative and sensitive as well (Fitria, 2018; Fitri et al., 2020; Nur et al., 2020). Subsequently, experiencing anxiety, anger, and depression can be reduced (Craft and Perna, 2004; Lotan et al., 2005; McKinty, 2013) meaning positive effects on health and well-being when a person engages in playing (Tolks et al., 2019).

Games, which are played in the natural environment, contribute to children’s social development and have a positive impact on their emotional intelligence, reinforce interpersonal relationships, and increase levels of happiness and emotional well-being (Kovačević and Opić, 2014; Bazaz et al., 2018). Moreover, games which are played with more than one player tend to create a sense of social interaction, emotional satisfaction, and flexibility and at the same time they enable players to develop a sense of discipline and conformity to rules (Lestari and Prima, 2017; Fitri et al., 2020). At the same time, they offer children the opportunity to experience a variety of emotions, sense of accomplishment, and cultivate a positive character (Aisyah, 2017; Tolks et al., 2019), since traditional games can contribute to the diminution of antisocial behaviors (Fitri et al., 2020).

Bazaz et al. (2018), who examined the effects of traditional games in regard to social development and emotional intelligence in preschool children, concluded that these kinds of games help participants improve on their social and emotional development, a finding that is supported by Rombot (2017) as well. These kinds of games enable children and adolescents to develop a sense of “belonging” (Weiss and Smith, 2002; Agans et al., 2018; Nur et al., 2020). However, the significant impact of play is not limited only to childhood. In older ages, play is considered a kind of amusement, which increases well-being, social interaction (Howard et al., 2017; Nur et al., 2020), and quality of life in general (Woodyer, 2012; Rogerson et al., 2013; Gelisli and Yazici, 2015). It is not a coincidence that play is defined as a form of human activity that follows a person throughout their early childhood (Howard et al., 2017) and continues to follow them throughout their life (Kovačević and Opić, 2014; Nur et al., 2020).

### Emotions and Type of Game

In regard to the nature of games, there are studies that have extensively examined the emotions experienced, taking into consideration the type of game (psychomotor or sociomotor/co-operative, opposition, and co-operation opposition) where sometimes the element of competition was measured as well (Alcaraz-Muñoz et al., 2020). The “specific state of affairs” of each game is also a specific state of affairs in terms of social relationships; the traditional games are suitable to create new social relationships (Parlebas, 2020). When the source of this uncertainty is based on the motor communication that takes place between players, four different motor domains can be established: (a) the psychomotor domain, in which the participant performs the task alone without any motor communication with others, as in a sac race competition and frog; (b) the cooperative domain, in which participants share a common language involving clear messages of assistance, as in the schini and mantili game; (c) the opposition domain, in which participants share a language of confusing signs, of messages that hide their true intentions, thereby enabling them to deceive their adversary, as in the case of ziziros; and (d) the cooperation-opposition domain, characteristic of those traditional games that combine opposition between rivals with collaboration among teammates as in schini and skoupa (Lavega et al., 2014). For example, the game of sakoulodromies – sack races (psychomotor plus competition) may produce joy in students, depending on whether they have won. The game of Schini (Rope) is a game cooperation–opposition with competition, because it is a game with having people in the different team and in the same team. In the game of ziziros (opposition without competition), they may experience joy when tagging another player, whereas the person who is tagged may feel anxiety (Lavega et al., 2014).

Competition and cooperation are elements that help participants interact with other people and have a more effective game (Cagiltay et al., 2015). It is considered to be a process that develops enthusiasm, motivates, and draws attention to the desire of a person to participate in a game (Cagiltay et al., 2015). In general, it was found that team games (sociomotor) cause more positive emotions than psychomotor (individual) games (Lavega et al., 2013) along with the element of competition when included. Through play, it is observed that it develops pleasure to succeed, especially when other people are there, strengthening individual responsibility (Lavega i Burgués and Navarro, 2015). Even though competitive games are characterized as more exciting, including the experience of more negative emotions when loss occurs, in case of winning, positive emotions outweigh the negative in terms of both frequency and quantity (Lavega et al., 2014). Besides, people who participate in such games tend to prefer social relationships related to competition (Lavega i Burgués and Navarro, 2015).

Summarizing, the experience of high levels of positive emotions and low levels of negative emotions constitutes a key aspect of emotional well-being (Houben et al., 2015). Taking as a fact that positive psychology emphasizes in subjective well-being and the role of flourishing, meaningful activities in any age, this could easily reinforce the argument that engaging in all kinds of such activities could lead to happiness in life (Waters, 2011).

### Categorization and Identification of Emotions

As previously mentioned, Seligman’s (2011) model includes positive emotions in regard to well-being meaning a group of emotions that have a positive impact on one’s well-being. Subsequently, on the other hand appears the category of negative emotions (make us feel discomfort/dysphoria) (Alcaraz-Muñoz et al., 2020). In order to study emotions, according to the literature, there are more ways in which they can be categorized, such as “primary emotions” (anger, surprise, fear, joy, sadness, and disgust) and “secondary emotions” (anxiety, irritation, aggression, rage, and hopelessness) (Leyens et al., 2001; Ednie, 2005).

Additional literature divides and categorizes emotions based on their intensity (Lavega et al., 2014, 2017; Alcaraz-Muñoz et al., 2020) and/or frequency (Martinent et al., 2012) as well. Hence, emotions that appear more in terms of duration and frequency could be stated that they prevail (to be frequent) under certain circumstances. The word “prevail” usually refers to something (in this instance, the emotions) that appears more frequently and lasts longer under the circumstances they appear.

Each emotion can be identified based on verbal and non-verbal expressions, such as facial expressions, gestures, posture, especially when identifying anger, sadness, and happiness (Coulson, 2004), and vocal cues (Ekman et al., 1971; Bassili, 1979; Juslin and Laukka, 2001; Sauter et al., 2010; Volkova et al., 2014; Wolf, 2015; Melzer et al., 2019). Furthermore, Alcaraz-Muñoz et al. (2020) refer to emotional scenarios which differ depending on the type of the emotion a player experiences, the intensity of that emotion, the personality and preferences of the player, and the rules of the game played (type of game).

## Purpose of the Study – Hypothesis

What inspired the implementation of this study was the Folklore Group «KTHMA» in Cyprus whose aim is to maintain and preserve the play of Traditional Cypriot Easter Games in Cyprus. Thus, the purpose of the current study was to study the emotional experiences of children and adults while taking part in the Traditional Cypriot Easter Games and how these experiences influence their emotional well-being, while focusing on the emotions that emerge while playing these games. This study is important to the literature as it appears to be one of the first studies to provide information on how different Traditional Easter Games are played in Cyprus nowadays, with an emphasis on the emotional well-being of the players.

Based on the purpose, the following hypotheses were developed:

H1: It is expected that Traditional Easter games will cause more excitement and euphoria in adults than children, as adults are expected to be more engaged during the games.

H2: In competitive games, more intense anger and frustration of defeat is expected to be observed.

### Research Design

#### Participants

The study used a qualitative analysis consisting of interviews and observations.

In the first part of the study, interviews with close-ended questions were carried out to 51 participants (32 men and 19 women) aged 32–93 years old.

In the second part of the study, audio-visual material was collected from people playing Traditional Cyprus Easter Games during Easter celebrations in April 2019. The sample consisted of 20 children, 6 girls and 14 boys, aged 6–14 years old (*M*_age_ = 10.45) and 43 adults, 12 women and 21 men, aged 18–65 years old (*M*_age_ = 42.16). Participants who took part in the second part of the study consisted of people who took part in the first part of the study and people from the surrounding areas who wanted to take part in the Easter Celebrations.

Demographic information and information on the various Traditional Cyprus Easter Games played in their villages were acquired by participants from 10 different villages around the Paphos district in Cyprus.

The study was reviewed and approved by the University’s Research Ethics Committee. All participants provided their written informed consent to participate in this study before the beginning of each part.

#### Procedure and Materials

The study was divided into two parts. In the first part (Part A), interviews were used to gather demographic information on participants as well as useful information on Traditional Cypriot Easter Games (i.e., what games were played, what games are still played, and description of each game). Gathering information on the games that were played and on the games that are still played will lead researchers to the second part of the study (Part B), which is the live demonstration of specific Traditional Cypriot Easter Games (based on information gathered form the interviews) in order to observe verbal and non-verbal expressions and body language of the participants while playing the particular games.

In the first part of the study (Part A), semi-structured interviews with close-ended questions took place with 51 adults, residents of the 10 villages included in the study. The semi-structured interviews were divided into two parts. Questions in Part A included personal questions to gather demographic data on participants [name, age (*M*_age_ = 58.86), sex, occupation, village of residence, and years of residence in their village (*M* = 52.55)]. Questions in Part B aimed to gather information on Traditional Cypriot Easter Games (i.e., what Traditional Cypriot Easter Games used to be played at each village and which of those games are played now and proved a description on each game name). The design was influenced by the gap that appears to be in the literature regarding information on Traditional Cypriot Easter Games. Each interviewee was interviewed on a one-to-one basis. The interviews were 20 min long, all interviews were audio-taped in order to avoid failing to record important information, and they were encoded in a later stage by the researchers. The researchers were responsible for carrying out the interviews, as they were equipped with adequate knowledge and experience to carry out the particular research tool. From the interviews, a list of 30 Traditional Cypriot Easter Games emerged; however, only 15 of them are still playing until nowadays.

The second part of the study (Part B) involved observations of live demonstrations of a number of Traditional Cypriot Easter Games that took place over Easter Sunday and Easter Monday in April 2019 during the First Panpafian Festival, as part of the Easter Celebrations. The village where the recordings took place was Pomos village square in Paphos. These games were recorded and visually studied by the researchers upon the completion of the games. Participants from the first part (Part A) of the study were also invited to participate in the second part (Part B) of the study. For the purpose of the study, it was decided to live demonstrate only 15 games with participants.

In addition to this, the researchers ended up analyzing only six of these games because of the way emotions were expressed (Coulson, 2004; Sauter and Scott, 2007). More specifically, the games that were chosen to be analyzed were those that clearly presented verbal and non-verbal expressions and body language of the participants. These games were Ziziros (Cicada), Schini (Tug-of-War), Vatrachos (Frog), Mantili (Handkerchief), Sakoulodromies (Sack Races), and Skoupa (Broom) (Appendix 1). In all of the games, apart from Skoupa (Broom), adults were grouped separately from children. The reason was the low participation on the side of female participants which made it difficult to create two groups based on age. It was not considered as a variable that could affect the study since all participants were either adults or adolescents with a small gap in their age range.

During the implementation of these games, both members of the Folklore Group and this study’s researchers were physically present. On the one hand, members of the Folklore Group along with the main organizers of the celebrations ensured that the festive celebrations were running smoothly. On the other hand, the researchers ensured that all games played were recorded (videos and photographs) accordingly in order to enable audio-visual observations to be studied afterward. Therefore, during this part of the study, researchers adopted a passive, non-instructive role, by ensuring that the recordings were going to take place. In order to ensure that all members of the team were in a position to undertake observations, they had to meet specific criteria. One of the members of the team, who is an academic at the University, is a qualitative researcher; therefore, it was her responsibility to ensure that the rest of the team met the specific criteria. These criteria included: speaking the same language and have the same culture as the participants, as well as be familiar with how the games that were going to be observed. Also, since the rest of the team were students at the University undertaking their MSc in Counseling Psychology, it was also mandatory to have successfully pass the specific course on qualitative research, as well as to have previous experience in participant observation through their training within their MSc. In addition to the above, all the team, before the beginning of the study, watched videos with games being played by adults and children that were uploaded in the internet and used the same observation lists that were going to use in this study in order to get more familiar with the procedure that was going to followed in the study.

Each game was going to be observed twice by all 5 researchers and every 30 s the observers were going to note on verbal, non-verbal expressions (facial expressions and vocal cues), body posture and gestures (Ekman et al., 1971; Bassili, 1979; Juslin and Laukka, 2001; Sauter et al., 2010; Volkova et al., 2014; Wolf, 2015; Melzer et al., 2019) being observed by writing them down. In order to establish reliability, observers had to be consistent. To achieve this, it was made clear that the observers were going to observe only what was important and relevant to the research. In other words, they were going to observe only the group of participants playing the games, ignoring any other behaviors expressed by people around. Another way to establish reliability was to be clear about what was going to be observed. All observers were going to observe verbal, and non-verbal expressions (facial expressions and vocal cues), body posture and gestures being presented only by participants playing the games. Verbal, and non-verbal expressions (facial expressions and vocal cues), body posture and gestures presented by participants who were not taking part in the game being observed, were going to be ignored. Also, in order to ensure that data gathered from observations were reliable, all five members of the research team were present during the observation analysis. All researchers, at the beginning, they observed the recorded videos twice in order to compos their own observation draft lists with expressions, body posture, and gestures presented by participants. These lists were created with the use of Excel program. After that, all five lists were put together, the researchers crossed checked and pointed out only the expressions, body posture, and gestures that were common in all five observation lists. Only these were going to be used in the final observation list and later on in the observation analysis. In this way, the researchers were able whether inter-reliability had been successfully introduced. In regard to intraobserver variability, the leader of the team, who also took part in the whole process, was able to observe material twice and her findings were in agreement with the rest of the team. It is also important to note that the age of participants and the type of game played were also taken into account. When choosing a game, one of the first variables to consider is the nature of its goal. Some games, like sac race, have the goal of winning. Other games have no inherent goal, and so, playing the game is an end in itself. In these “open” games, there are no winners or losers. Research has shown that the kind of goal can influence the emotional experience of participant (Lavega et al., 2014).

#### Data Analysis

To be able to decode demographic data gathered from the first part of the interviews, the IBM SPSS statistical analysis program was used.

In the second part of the study, a qualitative content analysis method was used in order to collect information on the emotional experience of the players while playing the games. As mentioned above, for the purpose of the study, only 15 out of 30 games were chosen to be played on that day and only six of them were analyzed.

For the purpose of this study, observations were employed in a descriptive way to identify different emotions presented while participants were playing the games. The observations were structured in order to enable researchers to gather information on different emotions expressed while playing the games. Taking into consideration the nature of the observations and their aim, which was to identify emotions that emerge during participants’ participation in the different games, a content analysis method of the observations was necessary to take place. This method enabled researchers to note and study the frequency of the different emotions expressed by participants before, during, and after the completion of each game.

Observation lists were used to identify the emotions presented by participants. Each researcher had collected his information with the help of observation lists. All five lists were crossed checked and created a final list with information about expressions, body posture, and gestures that were common in all of them. Then these information were identified with the corresponding feeling, creating a list of 17 emotions. Similar emotions were grouped together to identify the main emotions that best characterize participants experienced while playing the particular games. Some of these emotions appeared more than once (more frequent) or lasted longer than others. The analysis of our lists based on the frequency of the emotions appeared, served the purpose of the study which is to examine the effect of Traditional Easter games on the emotional well being. Hence, it was necessary to observe whether negative or positive emotions appeared more and lasted longer. Consequently, the two main characteristics the researchers based the configuration of this study were frequency and duration (Martinent et al., 2012) resulting in two main categories created exclusively for the purpose of this study, and named by the researchers as prevailing (appear more frequent and lasted longer) and secondary emotions (appeared less frequent and were shorter lasting). Distinguishing the emotions observed into these categories enables the identification of the prevailing emotions (appear more times) during the games (positive emotions = presence of well-being ⇎ negative emotions = absence of well-being).

The category of prevailing emotions includes joy, euphoria, excitement, impatience, anxiety, and satisfaction. Through the observation of the audiovisual material and the analysis of it, it occurred that these emotions are characterized as more intensive and lasting in terms of duration and frequency. The category of Secondary emotions includes frustration (to defeat), embarrassment, nervousness, anger, worry/restlessness/concern, and distress. Once more, through the observation of the audiovisual material and the analysis of it, it occurred that these emotions are characterized as less intensive and they last less in terms of duration and frequency (Sauter and Scott, 2007; Sauter et al., 2010).

### Emotion Determination

To begin with the prevailing emotions, joy was determined by facial expressions such as smiling, the intensity of their body movement, laughter, and happily shouting especially in moments of victory. Second, euphoria was a combination of positive emotions participants expressed throughout the games and was determined by the constant smile on their faces, their tendency to actively participate in the game and the general happy atmosphere that prevailed. Third, excitement was observed especially when someone won a game as well as when a player of a team made a good move, or gained a victory for the team. More specifically, participants were jumping up and down, clapping fast, yelling, and using expressions such as “Yey,” “Good job,” “Bravo, you did it!.” These expressions were characteristic in terms of the excitement expressed by the participants.

Fourth, impatience was determined by the intense body posture where the player was alert to participate or when his/her turn was coming up. Other body expressions that demonstrate this emotion included the back and forth movement of players and the use of expressions such as “Come on!,” “I’m next,” “When am I playing?,” and “Can I start now?.” Fifth, anxiety was observed through their hasty and nervous movements especially when there was a time limit in the game. Trembling of their limbs, fast breathing, nail and lip biting were also observed. Finally, satisfaction was verbally expressed with expressions such as “Yes!” in combination with the appropriate hand gestures, loud voice and was observed mostly when a player or a team-player was winning.


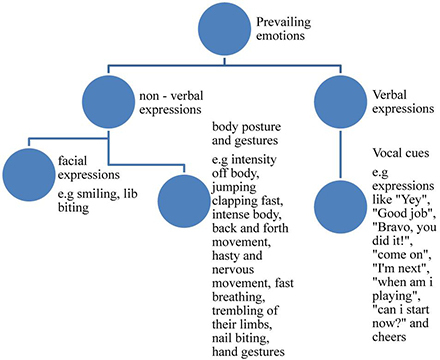


Regarding the secondary emotions, frowns on the faces of participants when they were losing, and less intense movement, were considered as indicators of frustration (of defeat). When experiencing embarrassment, participants and more specifically children, seemed more reserved or their body movements were more hesitant especially in moments when they did not know how to play the game or what to do next. Nervousness was expressed with lip biting and the need for fidgeting with their hands by playing with their hair and fingers. Anger was visible through facial expressions such as nose wrinkling combined with loud and intense yelling as well as intense foot moving (i.e., kicking) and hand clenching into a fist, especially when someone was losing or did something that cost the team the victory. Worry/restlessness/concern was observed by a mild intensity on the posture of the participants, when they were looking around asking what they should do or how the game is played. Generally, expressions and body movements were similar to those observed in anxiety but less intense and shorter lasting (instantaneously). Distress was a more intense form of anxiety that participants may have experienced during the games. It was the least observed, in matter of frequency, emotion and least intense of all the emotions observed. The moment when this emotion was expressed was when a player was disapproved by other team members or by the audience. In these cases, the participant, especially children, may have felt embarrassed as well, lowered his/her head, blushed, and behaved with hesitation while continuing to play.


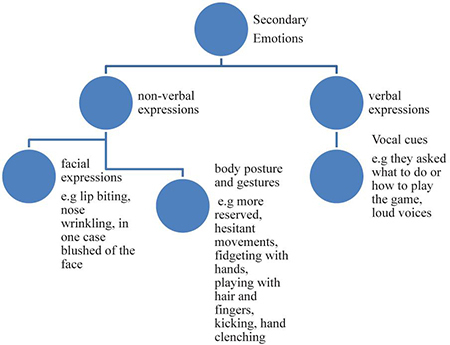


## Results

The results of the study concluded that only two games are still played today in all 10 villages: Ziziros (Cicada) and Rope (Tug-of-War). Nine games were played in the past: Afká tou Kolóka (Eggs of Kolokas), Vasiléas (The King), Vasilitziá tziai Myritziá (Basil and Myrtle), Kalispéra Afénti Goúmene (Good evening master Abbot), Koutsó (Hopscotch), Loukkoúin, Próti Eliá (First Olive), Pláka, and Triáppithkia (Jumping Three Times) (Table 1).

**TABLE 1:** Games played in how many villages (present and past).

	Game	No. of villages			Game	No. of villages	
					
		Present	Past			Present	Past
1	Avgoulodromíes (Egg Races)	7	9	16	Mantili (Handkerchief)	7	8
2	Afká tou Kolóka (Eggs of Kolokas)	0	2	17	Potamós (River)	5	9
3	Appíisen o Kámilos (Jumping Camel)	2	8	18	Próti Eliá (First Olive)	0	2
4	Vasiléas (The King)	0	2	19	Pláka	0	1
5	Vatrachos (Frog)	1	2	20	Dog and Bone	2	3
6	Vasilitziá tziai Myritziá (Basil and Myrtle)	0	2	21	Sakoulodromies (Sack Races)	9	10
7	Gaourodromíes (Donkey Races)	1	9	22	Sousa (Swing)	1	1
8	Gémisma tis stámnas (Filling the Pitcher)	1	1	23	Skoupa (Broom)	1	1
9	Ditzímin (Small Stones)	7	10	24	Skatoúlika (Small Stones)	4	10
10	Zíziros me Kapélo (Ziziros with a Hat)	1	7	25	Schini (Tug-of-War)	10	10
11	Ziziros (Cicada)	10	10	26	Sytziá (Fig)	2	3
12	Kalispéra Afénti Goúmene (Good evening master Abbot)	0	1	27	Kattómougia (Catfly)	1	9
13	Koutsó (Hopscotch)	0	2	28	Triáppithkia (Jumping Three Times)	0	9
14	Linkrín (Small Stones)	1	10	29	Treis énteka treis dódeka (Three eleven three twelve)	1	7
15	Loukkoúin	0	1	30	Faratsís	1	1

Vatrachos (Frog) and Treis énteka treis dódeka (Three eleven three twelve) were played in two villages though today they are both played in only one. Sakoulpdromies (Sack Races), Ditzímin, Linkrín, and Skatoúlika (Small Stones) were played in all 10 villages, while today they are played in nine, seven, one, and four villages, respectively. Avgoulodromíes (Egg Races) was played in nine villages and now only in seven. Appíisen o Kámilos (Jumping Camel) was played in eight villages though today it is played in only two. Gaourodromíes (Donkey Races) and Kattómougia (Catfly) were played in nine villages but today are played in only one. Gémisma tis stámnas (Filling the Pitcher), Soúsa (Swing), Faratsís were played and are still played today in only one village. Zíziros me Kapélo (Ziziros with a Hat), while in the past was played in seven villages, today it is only played in one. Potamós (River) was played in nine but today it is played now days in five villages; Skýlo kai kókkalo (Dog and Bone) was played in three and today in two villages and finally Sytziá (Fig) was played in only two of the 10 villages examined and continues to be played in two villages (Table 1).

While playing the first game, Vatrachos (Frog), participants experienced feelings of embarrassment, and anxiety. These emotions gradually increased to impatience, joy, and excitement. Adults compared to children experienced more frequently the emotions of joy, excitement, and euphoria from the start of the game. In this case, we observe that we expected that adults would feel these feelings more strongly than children (Tables 2, 3).

**TABLE 2:** Emotions observed in each game.

GAME	Emotions											
	
	Prevailing Emotions						Secondary Emotions					
		
	Joy	Euphoria	Excitement	Impatience	Anxiety	Satisfaction	Frustration (due to defeat)	Embarrassment	Nervousness	Anger	Worry/restlessness/concern	Distress
Vatrachos (Frog)	X		X	X	X			X				
Mantili (Handkerchief)	X	X	X	X			X				X	X
Sakoulodromies (Sack Races)	X		X	X								
Ziziros (Cicada)	X	X	X		X		X	X				
Schini (Rope)							X			X		
Skoupa (Broom)	X				X	X	X		X			

**TABLE 3:** Emotions in each game according to age group.

Game	Emotions	
	
	Adults	Children
Vatrachos (Frog)	More intensely the emotions of joy, excitement and euphoria from the start of the game	At first, they experienced emotions such as anxiety and embarrassment, but then they decreased and their position I got feelings of joy, impatience and excitement. Also, children compared to adults experienced less frequent feelings of joy, excitement and euphoria
Mantili (Handkerchief)	more intense emotions of excitement and impatience as compared to children even more excited and more impatient than children.	Experienced, higher anxiety, nervousness and embarrassment. Excitement was the lead emotion in this game, both for children and adults, as well as frustration for defeat.
Sakoulodromies (Sack Races)	Both children and adults experienced great joy and excitement experienced more excitement and joy, especially after winning the race.	Seemed to be more impatient right before the start of the game
Ziziros (Cicada)	X both children and adults experienced emotions of excitement, euphoria and joy	Anxiety, embarrassment and frustration were more commonly experienced by the main players, regardless of age
Schini (Rope)	Frustration was more intensive in both children and adults. Anger was also observed from participants, but in the sense of frustration for defeat both anger and frustration were observed after being defeated by the opposing team the winning team expressed more positive emotions, as expected, such as joy and excitement.	in children and adults it was observed how the feeling of frustration occurred more frequently, Also along with frustration, in individual moments the feeling of anger was observed for both age groups, especially if they were in the losing team
Skoupa (Broom)	The adults felt joy. When participants started to reduce the anxiety and nervousness appeared in both age groups. Frustration and satisfaction were feelings presented in a short period of time	Children felt the same joy as adults. As in adults so in children, when reduced the participants felt anxiety and nervousness for children frustration and satisfaction was presented the same as in adults

While playing the game Mantili (Handkerchief), emotions of excitement, frustration for defeat, euphoria, impatience, and joy were observed. Adults experienced more frequent emotions of excitement and impatience as compared to children. Children experienced worry, higher anxiety, distress, nervousness, and embarrassment. Excitement was the lead emotion in this game, both for children and adults, as well as frustration for defeat. It was also observed that adults were even more excited and more impatient than children (Tables 2, 3).

In Sakoulodromies (Sack Races), both children and adults experienced joy and excitement. Children seemed to be more impatient right before the start of the game and adults experienced more excitement and joy, after winning the race (Tables 2, 3).

In Ziziros (Cicada), both children and adults experienced emotions of excitement, euphoria, and joy. However, anxiety, embarrassment, and frustration were more commonly experienced by the main players, regardless of age (Tables 2, 3).

In Schini (Rope), frustration was more frequent in both children and adults. A momentary experience of anger was also observed from participants, simultaneously with frustration for defeat. Nevertheless, it is important to mention that both anger and frustration were observed after being defeated by the opposing team. On the contrary, the winning team expressed more positive emotions, as expected, such as joy and excitement. T his game is considered a competition, which is why anger and frustration (Tables 2, 3).

In the last game, the Skoupa (Broom), all participants experienced joy. During this game, as the number of participants decreased, the greater the anxiety and nervousness was observed in the remaining participants (Tables 2, 3). Frustration to defeat as well as satisfaction of winning the game was also observed. These emotions can be characterized as short lasting. There were not any differences in regard to the emotions experienced between adults and adolescents. There have not found age differences in ratings of intensity (Birditt and Fingerman, 2003). In their efforts to maintain close relationships, older adults may attempt to regulate their emotions in ways that minimize the intensity of problems (Birditt and Fingerman, 2003).

T hrough the description of the games, it turns out that in some games adults show more excitement and euphoria, but there are also games where there are no differences between the two age groups.

## Discussion

In this research, a variety of emotions were observed in participants through the audio-visual material collected. This wide emotional array was varying in frequency and duration. Emotions such as fun (joy), euphoria, and children’s anticipation of their turn to play again (impatience) indicate the range of positive emotions that the Traditional Cypriot Easter Games offer. An evidence supported by a study that examined the level of well-being male children experienced when participating or not participating in traditional games and found that children in the second category, reported distress, and stress problems (Gulia et al., 2020). Nonetheless, Traditional Cypriot Easter Games seem to entertain both children and adults.

### Emotions Experienced by Participants

Consistent with this research hypothesis, it was found that emotions such as euphoria and excitement were the most commonly positive emotions experienced in adults. For instance, Zizyros (Cicada) is a game that manifests emotions that enhance the well-being of the participants with adults displaying euphoria and joy when they participate in this game. Perhaps this finding is attributed to the fact that the Traditional Cypriot Easter Games were an opportunity for them to relive their childhood, feel free and escape from the responsibilities of adult life, reconnect with their roots and bring forth pleasant memories of that time, whereas the children’s notion was that they were participating in games their grandparents used to play as children. Additionally, it is interesting the fact that adults even temporarily cast aside their role as parents and enjoy the games like children. Normally, their parenting role drives them to regulate how, when and for how long their children will play baring in mind the safety and health of their children (Howard et al., 2017) something that has not been observed when they participated in the Traditional Games during the celebrations of Easter.

Adults were observed to be more competitive and enthusiastic (excitement) than children in the majority of the games, as well. It was evident that they were enjoying the experience and were dedicated to the game and to the whole process. The opportunity to play the same games as they did when they were children, consequently helps them reconnect with their childhood and the pleasant memories of that time since the context in which the games took place worked as an alibi for them to engage in playing, something they don’t consider acceptable to do as adults (Deterding, 2018).

### Adults’ and Children’s Emotional Experience

As for the differences, or similarities, between children and adults, it was observed that adults seemed to have more fun (joy) and displayed less negative emotions. Nevertheless, adults were once in the position that children are now, therefore they were already exposed to the games and they had the chance to experience them as well as experience the positive effects they offer (Gelisli and Yazici, 2015; Nur et al., 2020). This finding is in accordance with Vygotsky (1967) statement that adults are accustomed to adhere to the rules, which are present in every game. Adults are therefore able to be exposed to games and can easily adapt to them, because, as children, they learned to follow the rules given to them (Lestari and Prima, 2017; Fitri et al., 2020). Also, through participating in the game, the development of well-being, self-esteem, and the reduction of stress levels are observed in both groups (Treadaway et al., 2014).

### Emotions Experienced and Type of Games

The results of this study are also consistent with the second hypothesis. Through the analysis of the audiovisual material, it was noted that emotions change according to the nature of the game (Lavega et al., 2017; Alcaraz-Muñoz et al., 2020). When the type of result is loss, games with more competitive nature have been found to develop negative emotions more frequently (e.g., anxiety, anger) than non-competitive games under the same circumstances (Lavega et al., 2014). Of course due to the competitive nature of the game, when win occurred, more positive emotions were expressed.

On the other hand, the research also found that in the game Schini (Tug-of-War) that is still played today and has a competitive nature, healthy competition is kept alive. Simultaneously, observations revealed a moderate and momentary frustration due to defeat. The presence of frustration due to defeat could be justified by the competitive nature of the game and the type of result (winning team and defeated team) (Lavega et al., 2017). Meanwhile, the aspect of short lasting presence of this emotion could be explained by the fact that the context in which players participated was for fun. Consequently, the effect of defeat is minimized, since participation alone can cause positive emotions (Lavega et al., 2013).

Even though in their majority of emotions expressed were positive, in the game Mantili (Handkerchief) it was observed two negative emotions that were not observed in other games, worry and distress. Both of these emotions occurred when the player was unable to offer the victory to their team gaining their momentary disappointment. This observation has to do with the competitive nature of the game and the fact that this game belongs to the sociomotor category (team game) where the actions of the player affect the whole team.

### Limitations

The sample was studied through audiovisual material where observations on the emotions of children and adults were made. The results were analyzed through a qualitative content analysis method and all five researchers took part in the observations, audiovisual material was observed twice, and for the analyses the researchers used only the emotions that were in common in all observations lists. This way of analyzing minimized the risk of researchers being influenced by their subjective experience. A use of a questionnaire in this case would eliminate even more the possibility of observers being influenced. However, taking into consideration the nature of the study and the aim of the study which was to capture the emotions of participants while playing the games and analyze them, the qualitative content analysis method seemed to be the best method to be used, and as it appeared from the results, the research team was able to carry the study and continue with the analysis without any problems. This procedure would have been difficult to be carried out if a questionnaire was used in order to capture emotions expressed by participants while playing the games, when losing and when winning a game. These emotions can be captured best when observing participants while playing the different games, rather than completing a questionnaire before and after playing the games.

Therefore, future research could use methods such as self-report questionnaires and interviews to identify and measure the participants’ emotions about Traditional Cypriot Easter games.

Moreover, these emotions were observed in the cultural context of Cyprus, therefore, it is uncertain if the findings of this study can be generalized to reflect other cultures as well, even though research suggests that basic emotions are universal (Sauter et al., 2010). Future research may explore the universality of different cultures’ emotions, about traditional games.

#### Future Research

The differences in emotions between the two sexes could be compared more extensively, because research indicates that emotions are different between the genders when participating in games (e.g., men are more competitive and display anger upon defeat) (Lavega et al., 2014). Women express more positive emotions in games which are based on social relationships. Furthermore, women express less negative emotions in competitive games as compared to men. We could also refer to the effects that modern games (video games) have compared to traditional games.

## Conclusion

In recent years, there has been an increased amount of observations in the scientific literature that indicate the benefits of the Traditional Cypriot Easter Games as well as their contribution to emotional wellness. However, the present study is innovative, since it is the first study that provides information about the Traditional Easter Games of Cyprus, focusing on the emotional well-being of the participants. In other cultures, studies have been done in the past to investigate the effect of traditional games on an individual’s emotional well-being, but this is the first to be conducted in Cyprus.

It can be concluded that Traditional Cyprus Easter Games will constitute a valuable treasure for the future generations to come and a valuable attribution to the understanding of society since traditional games and their cultural identity are characterized as “the mirror of society” (Nefil et al., 2021). For instance, as it was seen in the game Skoupa, which was played only by women of different ages, characterizing the winner as the “best housewife” a fact that reflects the role women had in the earlier ages. Through the study of such cultural games, we can gain a deeper understanding of different societies and their deeper values that govern them. By studying them and perpetuating them, the tradition of Cyprus will be preserved and, at the same time, a different way of entertainment, socialization, and understanding as well, will be established, through the traditional games, both for adults and children.

## Data Availability Statement

The original contributions presented in the study are included in the article/supplementary material, further inquiries can be directed to the corresponding author/s.

## Ethics Statement

The studies involving human participants were reviewed and approved by the Human Research Ethics Committee – Neapolis University Pafos. Written informed consent to participate in this study was provided by the participants’ legal guardian/next of kin.

## Author Contributions

All authors designed the study, conceived the manuscript, conducted data analysis, wrote the drafts, and edited the manuscript.

## Conflict of Interest

The authors declare that the research was conducted in the absence of any commercial or financial relationships that could be construed as a potential conflict of interest.

## Publisher’s Note

All claims expressed in this article are solely those of the authors and do not necessarily represent those of their affiliated organizations, or those of the publisher, the editors and the reviewers. Any product that may be evaluated in this article, or claim that may be made by its manufacturer, is not guaranteed or endorsed by the publisher.

## Appendix

**TABLE A1:** Game descriptions (Papanikolaou, 2017).

GAME	DESCRIPTION
**Ziziros (Cicada)**	
A (main) player stands with their left palm open under their right armpit while their right hand covers their right eye so that they cannot see the other players who stand a short distance behind them. Another player is chosen to slap the main player’s left palm. The main player immediately turns around trying to guess which player slapped them. At the same time, all the other players attempt to deceive the main player by raising their right index finger and making the sound of the cicada “zzzzzzzzz”. If the central player discovers the culprit, he returns to his place in the group, otherwise he remains in the same position and the same procedure is repeated until they accurately identify the slapper.	
**Schini (Tug-of-War)**	
Two teams are formed. Each team stands and holds each end of a long and thick rope marked on the middle of its length with a sign. The floor is marked with a line aligned with the sign on the rope. A referee gives the mark to start the game saying “GO” and each team pulls their end of the rope. The team who wins is the one that manages to pull the other team towards them beyond the set point.	
**Vatrachos (Frog)**	
The rules of this game are the same as any other race game with the only difference that contestants need to frog-jump their way to the finish line. A frog is drawn on the ground with a line in the front and contestants need to stand behind that line. The game finishes when the first contestant reaches the finish line. Traditionally, only single and married men played this game, a tradition which is continued still today.	
**Mantili (Handkerchief)**	
Two teams are created with the players standing in semi circles. Teams stand opposite to each other leaving enough space in the middle. The referee stands in the middle of the field holding a handkerchief up. Then, each team choses a player who, on the referee’s mark, has to run and pick the handkerchief before the other player does. Then the one who did not catch it, chases the other. If they catch them before they make it to their team, they win, otherwise the player who catches the handkerchief gives the victory to their team.	
**Sakoulodromies (Sack Races)**	
Each participant takes a sack, places his feet inside holding the sack on top and stands in front of the starting line. On the referee’s mark they start to jump while wearing the sack. The goal is to finish the race first without losing their balance. In case that multiple races are held, winners of each round compete again together in order to get the top winner of all races. Usually the first three players compete again This game can be played with at least two players but the ideal number of contestants is 10 to 15.	
**Skoupa (Broom)**	
This game is played by both single and married girls/women in order to find out who is the best housewife. Participants form a circle and one of them holds a broom. As soon as the traditional music starts they quickly pass the broom to each other until the music stops. Then, the participant holding the broom when the music stops, leaves the circle. The game is repeated until only one participant is left. Then, the winner, in addition to the prize, also wins the broom.	
